# Chronic disease self-management in heart failure: A narrative review of performance gaps and emerging solutions

**DOI:** 10.1097/MD.0000000000046819

**Published:** 2025-12-26

**Authors:** Sanjana S. Nelogal, Sai Teja Yedam, Srija Reddy Koppula, Hafsa Imtiaz, Pranay Shettywarangale, Basira Shah, Vadali Avinash, Akshita Bhalla, Sweta Sahu, Tirath Patel

**Affiliations:** a Internal Medicine, Salmani Hospital, Ranebennur, India; b Internal Medicine, Teaching University Geomedi, Tbilisi, Georgia; c Internal Medicine, Kakatiya Medical College, Warangal, India; d Department of Medicine, Jinnah Sindh Medical University, Karachi, Pakistan; e General Practice, Kamineni Academy of Medical Sciences and Research Centre, Hyderabad, India; f Internal Medicine, Fatima Jinnah Medical University, Lahore, Pakistan; g Internal Medicine, Dr. Pinnamaneni Siddhartha Institute of Medical Sciences and Research Foundation, Vijayawada, India; h Internal Medicine, Punjab Institute of Medical Sciences, Jalandhar, India; i Internal Medicine, JJM Medical College, Davanagere, India; j Internal Medicine, Trinity Medical Sciences University School of Medicine, Saint Vincent and the Grenadines, Kingstown.

**Keywords:** chronic disease management, evidence-based practices, health outcomes, healthcare access, healthcare disparities, healthcare systems, heart failure, medication adherence, patient education, self-management

## Abstract

Heart failure remains a major global health challenge, and despite therapeutic advances, chronic disease self-management continues to show significant gaps at patient, provider, and system levels. This narrative review synthesizes evidence published between 2000 and 2025 across randomized trials, systematic reviews, guideline statements, and qualitative studies to examine the effectiveness of education, lifestyle modification, medication adherence strategies, digital health tools, and psychosocial support in improving self-management outcomes. The literature consistently demonstrates that patient education, structured exercise, and dietary modification enhance clinical and quality-of-life outcomes, while digital health interventions offer promising but variable long-term benefits. Persistent disparities rooted in socioeconomic status, cultural beliefs, health literacy, and access to technology influence the uptake and effectiveness of self-management strategies. Implementation science underscores challenges related to cost, scalability, and sustainability, whereas patient perspectives emphasize the need for simplified treatment regimens, caregiver involvement, and culturally tailored education. Overall, optimizing chronic disease self-management in heart failure requires a holistic, patient-centered approach that integrates evidence-based strategies with real-world implementation considerations and equity-focused adaptations. Future research should prioritize digital health innovations, caregiver-patient dyadic models, and culturally adapted interventions to reduce disparities and improve long-term outcomes.

## 1. Introduction and background

Heart failure (HF), as defined by the American Heart Association (AHA) and the American College of Cardiology (ACC), is “a complex clinical syndrome that results from any structural or functional impairment of ventricular filling or ejection of blood.”^[1]^ Also, Ponikowski et al in the European Society of Cardiology (ESC) define HF as “a clinical syndrome characterized by typical symptoms (breathlessness, ankle swelling, and fatigue) that may be accompanied by signs (elevated jugular venous pressure, pulmonary crackles, and peripheral edema) caused by a structural and/or functional cardiac abnormality, resulting in a reduced cardiac output and/or elevated intracardiac pressures at rest or during stress.”^[2]^

Ischemic heart disease is the leading cause of congestive heart failure.^[3]^ Ischemia (coronary artery disease, coronary dissection, and coronary embolism), valvular diseases (rheumatic heart disease and degenerative valvular disease), hypertension (both heart failure with reduced ejection fraction [HFrEF] and heart failure with preserved ejection fraction [HFpEF]), and primary cardiomyopathies are some of the causes of HF.^[4]^ Hypertrophic cardiomyopathy, arrhythmogenic cardiomyopathy, left ventricular noncompaction, mitochondrial myopathies, and ion-channel disorders (long QT syndrome, Brugada) are all genetic causes.

Acquired causes of cardiomyopathies include tachycardia-induced cardiomyopathy, peripartum cardiomyopathy, stress-induced cardiomyopathy (Takotsubo), substance abuse-induced cardiomyopathy (alcohol, cocaine), and toxin-related cardiomyopathies, such as those caused by anthracycline. Inflammatory conditions, such as myocarditis, Chagas disease, HIV, viral infections, and giant cell myocarditis, can also contribute to the development of cardiomyopathy. Secondary cardiomyopathies can arise from a variety of conditions, including amyloidosis, sarcoidosis, storage diseases like hemochromatosis and Fabry disease, and connective tissue disorders such as scleroderma. Thyroid disease and endomyocardial fibrosis are also significant causes. Nutritional deficiencies, such as a lack of selenium, beriberi, and kwashiorkor, can lead to anemia and cardiac complications. Arteriovenous fistulas, congenital heart diseases, and pericardial diseases are other notable contributors to secondary cardiomyopathy.^[4]^

Patients with stable chronic disease frequently modify their daily activities so that they show no or minimal signs of overt HF. Triggers for acute decompensation may include medication noncompliance, use of NSAIDs, increased intake of salt, or a recent infection.^[5]^ The most common presentation of an acute exacerbation of HF is shortness of breath, which is either exertional or orthopneic, and other accompanying symptoms, though not uncommon, include chest pain, lack of appetite, and fatigue with exertion. Patients with orthopnea may also develop a cough, especially when recumbent.^[4]^

The New York Heart Association (NYHA) functional classification classifies HF based on the severity of presenting symptoms, while the AHA/ACC classification combines clinical symptoms of HF with the presence of risk factors linked to the development of HF (Table 1).^[1]^

**Table 1:** Classification of heart failure.

American Heart Association and American College of Cardiology (AHA/ACC)^[1]^	• Stage A: Individuals in this group are at-risk for HF but have no symptoms, structural heart disease, or cardiac biomarkers (indicative of stretch or injury) • Stage B: Pre-HF, that is, no symptoms or signs of HF and evidence of one of the following: structural heart disease, evidence for increased filling pressures, or patients with risk factors and increased levels of BNPs or persistently elevated cardiac troponin • Stage C: Symptomatic HF, that is, individuals have a previously diagnosed structural heart disease with either a past medical history of HF symptoms or current symptoms • Stage D: Advanced HF, that is, marked HF symptoms that interfere with activities of daily living and with reiterative hospitalizations regardless of attempts to optimize guideline directed medical therapy (GDMT)
New York Heart Association (NYHA) Functional Classification^[5]^	• Class I: Symptoms presenting with exertion beyond the normal level of activity • Class II: Symptom onset with normal level of activity • Class III: Symptom onset with minimal activity • Class IIIa: No dyspnea at rest • Class IIIb: Recent onset of dyspnea at rest • Class IV: Symptoms at rest
Left ventricle ejection fraction^[1,4]^	• HF with reduced ejection fraction (HFrEF): LV EF ≤ 40% • HF with mildly reduced ejection fraction: LV EF 41% −49% and evidence of HF (elevated cardiac biomarkers or elevated filling pressures) • HF with preserved ejection fraction (HFpEF): LV EF ≥ 50% and evidence of HF (elevated cardiac biomarkers or elevated filling pressures) • HF with improved ejection fraction: LV EF > 40%, with previously documented LV EF ≤ 40%

ACC = American College of Cardiology, AHA = American Heart Association, BNP = B-type Natriuretic Peptide, GDMT = guideline directed medical therapy, HFpEF = heart failure with preserved ejection fraction, HFrEF = heart failure with reduced ejection fraction, LV = left ventricle/left ventricular, NYHA = New York Heart Association.

### 1.1. Prevalence

Experts define HF as a global epidemic, impacting over 64.3 million individuals globally.^[6]^ In developed countries, the prevalence estimates range from 1% to 2% of the general adult population.^[7]^ HF is mainly a disease of older individuals, with a prevalence of 5% to 9% in people over and above 65 years. A meta-analysis using echocardiographic studies as a metric for screening the general population revealed a prevalence of “all-type” HF to be 4.2% in the general population and 11.8% in people over and above 65 years of age. Up to 76% of missed HF diagnoses are of HFpEF, indicating an unrecognized increase in HFpEF.^[8,9]^ The absolute number of patients living with HF worldwide has been increasing because of the aging population, global population growth, and improved survival after diagnosis.^[7]^ Apart from Sub-Saharan Africa, most of the developing countries are seeing an increase in mortality due to noncommunicable, degenerative, and chronic diseases like HF.^[3]^

In recent years, the age-adjusted incidence of HF has mostly followed a downward trend or is unaffected, but the overall incidence is increasing due to aging.^[10,11]^ Mortality and hospital admissions have remained steady, reflecting an increase in disease burden. This is potentially due to an increase in patient age, prevalence of risk factors, gaps in preventative care, and ineffective treatment strategies, especially concerning HFpEF.^[11]^ Women have a lower incidence of HF, but they still constitute approximately 50% of the prevalent cases. HFpEF is more common among females.^[7]^ Age is a major risk factor for HF development, and 1 in 3 people over 55 will develop the disease in their lifetime.^[12]^

### 1.2. Impact of heart failure on patients’ quality of life and healthcare systems

By 2030, estimates suggest that the prevalence of HF in America will exceed 46%, with over 8 million Americans over 20 years of age expected to develop HF.^[13]^ With the disease on the rise, the impact on quality of life (QoL) and the cost of healthcare are areas of major concern for the patients, their families, and healthcare policymakers.^[14]^

In congruence with the rise of disease prevalence, medical costs will increase from $20.9 billion in 2012 to $53.1 billion in 2030, reflecting a 250% increase in disease prevalence. Hospitalizations account for 80% of these cost increments. The indirect expenditure (reflective of morbidity and mortality) will increase from $9.77 in 2012 to $16.64 in 2030, representing an increase of 69%.^[15]^

The definition of QoL for either chronic or acute decompensated HF is unclear. Depression and disrupted social functionality are known to have a significant impact on health-related QoL in HF patients, though they are often underrepresented. QoL is affected by things like pulmonary congestion, reduced perfusion leading to myopathy and decreased renal blood flow, activation of inflammatory and neurohormonal pathways, and right ventricular impairment. All of these things happen in the context of severe hemodynamic compromise, which causes muscle loss and a cachectic habitus. QoL is known to worsen with increases in NYHA functional classification. Exercise intolerance also correlates with poor QoL.^[16]^ Multiple studies examining whether QoL is associated with predicting mortality are not reliable. Some studies, however, have found an association between poor QoL and deterioration in survival.^[17]^

Since clinical examinations cannot assess patient experiences, perceptions, and expectations about their disease process, QoL has emerged as an important metric in HF treatment strategies. The Minnesota living with heart failure questionnaire (MLHFQ) is a frequently used health-related QoL questionnaire for patients with HF,^[14]^ and others, such as the Kansas City cardiomyopathy questionnaire (KCCQ), include 3 major categories, physical, social, and emotional.^[16]^

As with many chronic diseases like diabetes, COPD, and asthma, the treatment of HF also includes various disease management programs based on the chronic care model model of Wagner^[18]^ that include components like self-management support, clinical information systems, and decision support. Numerous studies conducted to assess the cost-effectiveness of these interventions reveal a varying pattern, with some showing an increase in health costs due to direct healthcare expenditures ranging from $4970 to $3305 per patient per year, while others report cost savings through reduced hospitalizations and emergency room visits. The effectiveness of disease management interventions varies significantly, as evidenced by the wide range of costs per quality-adjusted life year gained, ranging from $17,747 to $1,56,655.^[19]^

### 1.3. Importance of chronic disease self-management (CDSM)

The growing impact of chronic diseases on healthcare and society has raised significant concerns. Addressing this challenge involves shifting toward patient-centered approaches, with self-management interventions being a key component.^[20]^

Self-management refers to “the ability of the individual, in conjunction with family, community, and healthcare professionals, to manage the symptoms, treatments, lifestyle changes, and psychosocial, cultural, and spiritual consequences of health conditions are all considered aspects of self-management.^[21]^ Self-management encompasses a variety of processes, such as setting goals, gathering and processing necessary information, evaluating this information, making decisions, taking actions, and responding to oneself.^[22]^

It involves collaborative healthcare where patients work closely with professionals. Unlike traditional healthcare, self-management requires collaboration; it considers the patient’s specific goals, and professionals assist in achieving the goals by providing informed choices. Collaborative care adapts problem-solving strategies based on patient goals, while traditional care may not align problem-solving with patient objectives and often attributes it to noncompliance. Patient behavior in traditional care relies on external motivation, while collaborative care emphasizes internal motivation with professional support.^[23]^

Patients benefit greatly from CDSM, a modern science that enhances their QoL, reduces their use of healthcare resources, and improves their overall clinical outcomes. CDSM constructs agreed plans between patients and health professionals, increasing self-efficacy and decreasing health distress.^[24]^ The ESC recognizes self-management as integral to achieving optimal patient outcomes in congestive heart failure. One of the core components of HF management is self-management, which includes domains like self-care maintenance, monitoring, and self-care management.^[25]^

Self-care maintenance involves patient practices and behaviors to maintain physical and emotional stability. Self-care monitoring involves tracking changes related to HF, such as changes in weight. Self-care management involves specific actions in response to a change in symptoms or signs of HF, like the titration of diuretics with the development of edema and weight changes.^[25]^

Patients with HF face challenging and infuriating responsibilities for self-management. These tasks include daily weight and symptom monitoring and analysis, as well as adjusting medication and behavior based on symptoms.

Various factors affect self-management in patients with HF. Internal factors include cognitive ability, health literacy, behavior change, and self-efficacy. External factors are knowledge, skill, socio-economic, therapy-related factors, healthcare team factors, and health system factors. The 2021 ESC recommends tailoring the organization of follow-up interventions to the individual patient’s needs and disease stage.^[26]^

### 1.4. Conceptual/theoretical framework for heart failure self-management

**Figure 1.**
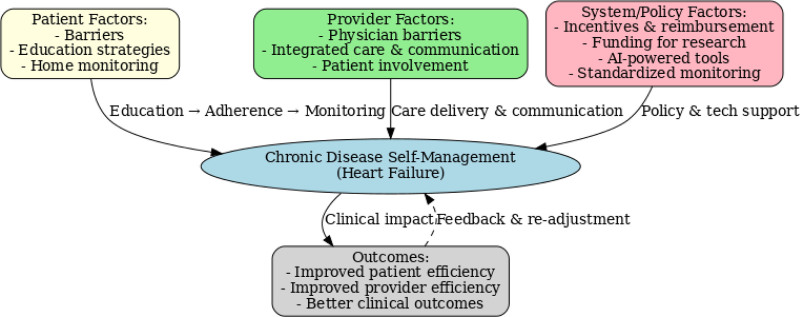
Conceptual model of chronic disease self-management in heart failure.

Figure 1 shows how patient, provider, and system factors interact to drive HF self-management. At the patient-level, education, motivation, and health literacy enable adherence and self-monitoring; provider inputs sustain engagement; and system elements such as care coordination, digital infrastructure, and community resources support implementation. Bidirectional arrows indicate feedback loops, which in turn reinforce adherence and trust. Monitoring data also informs provider decisions, prompting treatment adjustments that optimize results. Overall, the model moves beyond a linear view to emphasize continuous, interdependent relationships that, when aligned, improve QoL and reduce hospitalization.

There is a lack of sufficient evidence on how to use various models of self-management in HF. Various hurdles are present at different levels of care, including the patient level, physician level, and system level, hindering the self-management of HF.

The primary purpose of this article is to identify the core reasons for the suboptimal performance of chronic self-management treatment strategies in the treatment of HF, and to discuss any performance gaps in CDSM for HF. Identification of these gaps will help us to fine-tune our approach to being able to derive real-life and measurable improvements in the treatment of HF. The findings will aid in enhancing patient outcomes, providing enhanced and ongoing care, optimizing resource utilization, reducing hospitalizations, empowering patients, formulating healthcare policies and legislation, and reducing health care disparities.

## 2. Materials and Methods

This article is a narrative review synthesizing the current literature on chronic disease self-management (CDSM) in HF. Unlike a systematic review, our aim was not to exhaustively capture every publication but to identify recurring themes, evidence gaps, and implementation challenges across multiple domains.

We searched PubMed, Embase, and Google Scholar from 2000 to May 2025 using combinations of the following keywords: HF, self-management, patient education, telehealth, mobile health (mHealth), lifestyle modification, disparities, adherence, psychosocial support. We included English-language, peer-reviewed articles, major guideline documents, randomized controlled trials (RCTs), systematic reviews, and selected qualitative studies relevant to patient experience. Grey literature and non-peer-reviewed sources were excluded.

Two authors independently screened titles and abstracts for relevance, and disagreements were resolved by consensus. The focus was on extracting themes and insights rather than performing formal meta-analysis.

### 2.1. Ethics approval

Not applicable, as this study is a narrative review of published literature and did not involve human participants or patient data.

## 3. Review

### 3.1. Components of chronic disease self-management for heart failure

#### 3.1.1. Patient education

Knowledge plays a crucial role in HF patients’ survival. It empowers them to make lifestyle adjustments and practice self-care. These changes have an impact on reducing deaths and hospital returns. Many patients with this condition face these risks without a proper understanding. Gaining insight into their condition proves vital for better outcomes.^[27,28]^

Researchers developed a 20-item tool, known as the Heart Failure Knowledge Scale (HFKS), to assess knowledge about HF in a study. Analysis of this scale has revealed that a significant minority of patients were unclear about the symptoms to expect with worsening of their HF. While >90% of patients recognized some worsening HF symptoms (such as fatigue, shortness of breath, and limb edema), many failed to identify others (like ascites or abdominal swelling, needing extra pillows to sleep, and weight gain), with 52%, 41%, and 22% of patients, respectively, missing these symptoms. Furthermore, many patients mistakenly identified non-HF-related symptoms, such as neck pain or slurred speech, as indicative of HF. Although 91% of patients understood the importance of keeping a daily diary of weight and symptoms, nearly one-third (30%) believed that HF patients only needed to weigh themselves once a week, and a similar proportion (31%) did not recognize hypervolemia (described as “too much fluid”) as a cause of worsening HF symptoms. One-third of the patients believed weighing themselves once a week was adequate for assessing fluid status, and a similar proportion did not grasp the role of increased intravascular volume in HF exacerbations, highlighting the importance of thoroughly assessing patients’ beliefs about self-care practices as well as educating them.^[29]^

Knowledge of HF doesn’t ensure patients will follow all necessary behaviors. Yet, this understanding plays a crucial role. It allows patients to be part of their own care. It also helps them stick to the intricate medical plan. This adherence proves vital in warding off flare-ups of their condition.^[30]^

#### 3.1.2. Common educational interventions and their effectiveness

Patient education is a top priority in the nonpharmacological management of chronic diseases like HF.^[31]^ It is a crucial component of managing chronic illnesses and can enhance patients’ HF knowledge and self-care.^[31,32]^ Therefore, effective health education is vital, but it must also be accessible and user-friendly. Proper education helps patients with HF, understand, adapt to, and manage their condition.^[33]^ The rise of telehealth represents a significant advancement in 21st-century healthcare, offering a modern approach to delivering optimal care.^[34]^ Telehealth can provide tailored patient education through advanced communication technologies and user-friendly devices like smartphones and tablets.^[35]^ This method has proven effective in managing chronic diseases and has permeated all healthcare settings, greatly enhancing patient health and well-being.^[33,36]^ mHealth, a subset of telehealth, helps overcome health literacy barriers by providing health information.^[33]^ It plays a crucial role in improving health status and well-being, enhancing patient knowledge, promoting adherence to therapeutic management, and boosting self-care.^[37]^

mHealth education applications can enhance patient self-care management by providing timely, accessible, and cost-effective information. Patients often forget or misplace the information they receive verbally or in writing at discharge.^[33]^ A 2016 global survey by World Health Organization highlighted the importance of telehealth in managing various chronic diseases to improve the quality, cost, and accessibility of healthcare services.^[38]^ Outpatients can manage HF by providing proper discharge planning instructions, promoting a healthy lifestyle, and preventing readmissions.^[39]^ The study’s findings suggest that using a mobile application as an educational tool can improve patients’ knowledge, enhance self-care management, and support adherence to the recommended regimen. Increased knowledge empowers patients to adopt healthy behaviors, manage their health effectively, maintain daily activities, and improve their physical and emotional well-being.^[40]^

Another method is Teach back, which involves asking patients to repeat the information they have learned. It effectively enhances patients’ comprehension of HF and chronic illnesses. It is also an effective educational strategy for improving self-care and self-efficacy in HF patients.^[41]^

Establishing a WhatsApp chat group, which fosters friendship, information exchange, and communication among patients, aids comfort, confidence, enhances functional status, and promotes self-care, a phenomenon that support groups have demonstrated, necessitates further research.^[42–44]^ This method is more suitable for HF patients who have busy jobs and are comfortable using these communication tools.^[45]^

### 3.2. Lifestyle modifications

#### 3.2.1. Dietary recommendations

Plant-based foods such as fruits, vegetables, seeds, legumes, and nuts appear to be beneficial in the prevention of HF and atherosclerosis, with a lower consumption of processed foods and animal-based foods. On the other hand, diets high in phosphatidylcholine, which is present in red meat, cheese, and eggs, appear to increase the levels of trimethylamine-*N*-oxide in the intestines, a condition associated with an increased risk of heart attack, stroke, HF, and death. Higher fruit and vegetable intake is associated with better cardiovascular outcomes.^[46–48]^

Adherence to the DASH diet, which consists of foods low in saturated fat and high in fruits and vegetables, whole grains, beans, and legumes, is associated with decreased mortality among women with HF. It is also linked to improved left ventricle/left ventricular function in a multiethnic population without overt cardiovascular disease.^[49]^ In HFpEF, it is associated with beneficial changes in diastolic and arterial function.^[50]^ More closely, following the DASH diet has been linked to a lower risk of HF^[51]^ and a lower level of N-terminal pro-brain natriuretic peptide.^[52]^ A 20% improvement in diet quality scores was associated with an 8% to 17% reduction in all-cause mortality.^[53]^

Sodium restriction is another dietary recommendation that can be beneficial to patients with HF. The optimal level of sodium restriction for patients at high risk of cardiovascular disease or HF remains unclear. The AHA advises limiting sodium intake to <1500 mg/d.^[54]^ Given the connection between sodium and blood pressure (BP), this recommendation applies to patients with stage A and B HF, many of whom have hypertension.^[55]^ For those with diagnosed HF, the 2010 Heart Failure Society of America guidelines suggest a daily sodium limit of 2 to 3 g.^[56]^ Despite their widespread acceptance, the data supporting these recommendations is inconsistent and controversial.^[57–59]^

Research has shown that limiting sodium intake is linked to worsening neurohormonal profile^[60–62]^ and a higher risk of heart or HF-related hospitalization^[63]^ in people with HF. Sodium restriction might be most beneficial for patients in the NYHA functional classes III and IV.^[64]^

The 2013 AHA/American College of Cardiology Foundation (ACCF) guidelines recommend sodium restriction (class IIa) for patients with symptomatic HF to alleviate congestive symptoms, but they also note the lack of strong evidence for this recommendation.^[55]^ Patients with self-reported HF have poor adherence to sodium restriction, with only 34% adhering.^[65]^

Coenzyme Q10 supplementation in HF has a positive effect on left ventricular ejection fraction, with or without improvement in NYHA functional class.^[66,67]^ Researchers have linked a deficiency in coenzyme Q10 to the severity of HF symptoms and the extent of left ventricular dysfunction^[68,69]^ as well as to higher mortality rates in HF patients.^[70]^ This is one of the few dietary supplements that have been studied in stages C to D of HF and has shown a decrease in HF hospitalizations, cardiovascular mortality, and an improvement in the NYHA functional class.^[71]^

#### 3.2.2. Guidelines for exercise and physical activity

Lack of physical activity and low cardiorespiratory fitness (CRF) are independent, modifiable risk factors for developing HF.^[72,73]^

There is a strong dose-dependent inverse relationship between physical activity levels and the risk of developing HF. For middle-aged individuals with low fitness levels (<8 metabolic equivalents), improving CRF is linked to a reduced risk of HF in later life, regardless of BMI.^[74]^ Similarly, independent of other risk factors, a slower decline in CRF is associated with better systolic and diastolic function, and higher physical activity levels are associated with a lower HF risk.^[75,76]^

Current guidelines suggest engaging in 30 minutes of walking at least 5 days a week, or a total of 2 hours and 30 minutes of moderate-intensity aerobic activity per week. Alternatively, they recommend 1 hour and 15 minutes (75 minutes) per week of vigorous-intensity aerobic exercise, or a combination of both moderate and vigorous activities, for optimal cardiovascular health.^[77]^

Exercise training is an effective treatment for both HFrEF and HFpEF. In HFrEF, it is associated with decreased mortality, but a greater reduction in the combined rate of all-cause mortality and hospital stays in women with HF compared to men.^[78,79]^ The prescription of exercise training presents challenges, including lower adherence after the initial supervised visits. According to ACC/AHA guidelines, patients with HFrEF strongly recommend cardiac rehabilitation or exercise therapy, which the HF-ACTION trial results have led the Center for Medicare and Medicaid to cover.^[55]^

Higher exercise intensity, beyond the recommended guidelines, reduces the risk of developing HF more than low-intensity exercise.^[80]^ It is especially important for high-risk groups, like elderly, frail HF patients with multiple comorbidities and symptoms that make it hard to do things, to do strength training to fight sarcopenia and inspiratory muscle training to improve functional capacity. Innovative methods like telemedicine, exercise systems, and behavioral coaches may boost adherence.^[81]^

#### 3.2.3. Smoking cessation and alcohol moderation

Cigarettes and alcohol can contribute to hastening the onset and progression of HF. There is evidence that moderate alcohol consumption leads to a lower incidence of HF, whereas heavy alcohol consumption can cause dilated cardiomyopathy. Patients with HF may benefit from improved detection and treatment of drug misuse, as substance abuse disorders are associated with worsening HF and hospitalizations.^[70]^

Advise patients to refrain from heavy and binge drinking. Smoking is linked to worsening outcomes in HF patients, while quitting is linked to a lower incidence of serious adverse cardiac events. There should be a strong focus on encouraging current smokers to quit smoking. Even a minor decrease in cigarette smoking shows a therapeutic advantage in patients with HF.^[67]^

### 3.3. Medication adherence

Numerous studies have highlighted poor medication adherence among HF patients, influenced by various factors.^[82,83]^ Studies have demonstrated favorable outcomes, a decrease in hospital admissions, and mortality in patients who have good adherence to medication.^[84]^ Several factors contribute to poor medication adherence in HF patients; these include insufficient knowledge about the disease, fewer frequent follow-up visits, ineffective communication between the doctor and patients, cultural beliefs, a lack of family support, inadequate referrals to rehabilitation after discharge, financial condition, limited access to health care facilities and rehabilitation, comorbid conditions such as vision disorders, and complexity of treatment.^[85,86]^ Enhancing treatment adherence requires several key approaches, including educating and training patients, ensuring access to health care, reducing costs, providing health information, promoting self-care, implementing physician-oriented strategies, leveraging technical solutions, and utilizing telemonitoring.^[87]^

#### 3.3.1. Symptom monitoring and management

HF patients can employ several strategies to prevent hospital admissions. Patients should actively monitor themselves for any new changes, such as an increase in body weight or the emergence of new symptoms.^[88,89]^ A multidisciplinary approach including education about HF, advice from dieticians, and counseling can enhance awareness among patients.^[88]^ Additional research into remote monitoring options, such as telemonitoring, which involves transferring physiological data such as BP, EKG changes, weight fluctuations, and oxygen saturation, has the potential to detect possible complications as soon as possible.^[88,89]^ In the future, implantable devices that continuously monitor patients’ physiological parameters could play a crucial role in symptom management for HF patients.^[89]^ Providing well-defined action plans enables patients to track their symptoms with ease.^[90,91]^

#### 3.3.2. Psychosocial support

Psychological distress in HF patients has an impact not only on medication adherence but also on outcomes.^[92]^ Research reveals that HF patients experience an increase in hospitalizations due to psychological distress, lower socioeconomic status, and a lack of support.^[92]^ Social support plays a vital role in improving emotional well-being, promoting medication adherence, and decreasing hospitalizations.^[93]^

### 3.4. Current performance gaps in CDSM for heart failure

#### 3.4.1. Patient-level gaps

##### 3.4.1.1. Knowledge and awareness deficiencies

HF poses a significant public health challenge worldwide, including in India. It is a complex syndrome characterized by structural and functional abnormalities of the heart, leading to symptoms such as dyspnea and fatigue. The condition’s progression can be unpredictable, and it manifests with clinical signs like increased jugular venous pressure. Recognized as a global pandemic, HF affects a substantial number of individuals, underscoring its importance in medical practice and public health initiatives.^[94]^

Because HF is a progressive and debilitating condition, it necessitates lifelong management to achieve therapeutic goals effectively. The primary objectives of managing HF include enhancing self-care practices, minimizing adverse effects, reducing hospital admissions, and enhancing overall QoL. Treatment guidelines advocate for a combination of medication and non-medication strategies to manage the disease. Non-medication approaches, such as self-care, play a crucial role in preventing recurrent hospitalizations and improving health outcomes. Consistent adherence to self-care empowers patients to better control their HF, enhancing their ability to perform daily activities and manage social interactions, thereby improving their QoL. Effective self-care practices are essential in mitigating the progression of HF and promoting positive clinical outcomes.^[95]^

Knowledge and awareness are essential aspects of chronic disease self-management (CDSM) for individuals with HF. Successfully managing the condition involves patients having a thorough understanding of their illness, which includes understanding the nature of HF, the importance of taking medications as prescribed, making necessary lifestyle adjustments, and knowing how to identify and address symptoms. Despite improvements in medical education and resources, many HF patients still face substantial gaps in knowledge and awareness. These gaps can hinder their ability to effectively manage their condition.

##### 3.4.1.2. Common deficiencies

Patients with HF often lack essential knowledge about several key aspects of their condition. Many patients do not fully understand what HF is, its causes, or its progression. This fundamental gap can lead to a lack of engagement in management practices. There is a global agreement that people often mistake the early signs and symptoms of HF for normal aging, leading to a late detection of the disease.^[96]^ In primary care, significant gaps in access to diagnostics cause disparities and delays in diagnosis. Consequently, we often diagnose HF late, once the disease has reached an acute stage.

##### 3.4.1.3. Medication adherence

Medication adherence is often overlooked in clinical settings, despite being a widespread issue. Patients frequently lack essential knowledge about how to take their medications, potential side effects, and the consequences of nonadherence. Medication-taking behavior is complex, and nonadherence cannot be reduced to a simple binary issue.^[97]^

To address this, clinicians should simplify medication regimens and encourage the use of blister packs to help patients manage their doses. Health systems should implement comprehensive, multidisciplinary interventions to tackle nonadherence. While various strategies have been effective, there is no one-size-fits-all solution, and both individualized and population-based approaches require further evaluation.

##### 3.4.1.4. Symptom recognition and management

In HF, symptom recognition and management are critical aspects of patient care that often suffer from a significant lack of awareness. Many patients and their caregivers are not adequately educated about the early signs of HF exacerbation, which can include increased breath shortness, sudden weight gain, swelling in the legs and ankles, and fatigue. Because these symptoms can be mistaken for other less serious conditions or simply attributed to aging, patients may not seek medical help promptly.

#### 3.4.2. Lifestyle modifications

Lifestyle modifications play a crucial role in managing HF. Key strategies include dietary changes, such as adopting a low-sodium diet to help manage fluid retention and reduce strain on the heart. A structured exercise program is also important, designed to improve cardiovascular fitness and overall heart function. Behavioral modifications, like smoking cessation and stress management, are essential for promoting heart health. Additionally, patient education is vital, ensuring individuals understand the importance of these lifestyle changes for long-term heart health and preventing exacerbations.^[98]^

These components represent a holistic approach to managing diastolic HF, with the goal of improving patient outcomes and enhancing QoL.

#### 3.4.3. Addressing knowledge and awareness deficiencies

To improve knowledge and awareness among HF patients, healthcare providers can implement several strategies.

##### 3.4.3.1. Implementing structured education programs

This is how structured education programs can be implemented. Studies have shown that in-person educational sessions significantly improve patient understanding and self-management skills in HF management. These sessions provide an opportunity for direct interaction with healthcare professionals, allowing tailored education that addresses individual patient needs, concerns, and comprehension levels. Research indicates that patients who participate in regular face-to-face sessions are more likely to adhere to treatment plans and effectively manage their condition. Online educational modules offer a flexible and accessible approach to delivering HF management information. Studies suggest that interactive online platforms can effectively engage patients, allowing them to learn at their own pace and revisit materials as needed. Incorporating multimedia elements such as videos and quizzes enhances patient engagement and knowledge retention, contributing to improved self-care practices and health outcomes. Printed educational materials, including pamphlets and booklets, play a crucial role in reaching patients who prefer or require physical resources. These materials provide comprehensive information on HF management, including treatment guidelines, lifestyle recommendations, and symptom recognition. Research supports the use of printed materials as effective tools for reinforcing key concepts discussed during clinical visits, promoting patient empowerment and adherence to therapeutic regimens.^[99]^

##### 3.4.3.2. Patient counseling

Patient counseling is a critical component of effective chronic disease management, particularly in conditions like HF. It involves structured interactions led by healthcare professionals such as nurses or pharmacists, aimed at empowering patients with knowledge and skills to manage their condition proactively.

For example, in nurse-led HF clinics, patient counseling plays a pivotal role in enhancing patient outcomes. By delivering personalized guidance, addressing patient concerns, and reinforcing crucial information on medication adherence, dietary management, and symptom recognition, nurses empower patients to take an active role in managing their condition. Research underscores the effectiveness of these counseling sessions in improving patient understanding and self-management skills, ultimately reducing healthcare utilization and enhancing QoL.^[100]^

##### 3.4.3.3. Use of technology

Leveraging mHealth applications and telehealth services has revolutionized patient education in chronic disease management, including HF. These technologies provide accessible and interactive platforms for continuous learning, remote monitoring, and timely support. By enabling patients to access educational resources, track their health metrics, and communicate with healthcare providers remotely, these tools promote adherence to treatment plans and improve health outcomes.

##### 3.4.3.4. Behavioral and motivational challenges

Behavioral and motivational challenges play a crucial role in shaping the ability of HF patients to independently manage their condition. These challenges encompass a variety of factors that affect patients’ behavior and motivation to follow treatment plans and make necessary lifestyle changes.

#### 3.4.4. Nonadherence to medications and treatment plans

Nonadherence to medications and treatment plans is a significant issue in HF management, with various contributing factors and serious consequences for clinical outcomes.^[97]^

##### 3.4.4.1. Contributing factors

Patients often struggle with multiple medications and dosing schedules. Adverse effects and doubts about medication effectiveness deter adherence. Depression, anxiety, and lack of social support undermine adherence. High costs lead to skipped doses or rationing. Insufficient understanding of HF and the importance of adherence.

Nonadherence has an impact. More frequent hospital admissions due to exacerbated symptoms. Greater mortality risk due to disease progression. Frequent and severe symptoms reduce QoL. More hospitalizations and interventions lead to higher overall healthcare costs.^[97]^

#### 3.4.5. Strategies for improving adherence

Improving medication adherence in HF management requires several targeted strategies. Simplifying treatment regimens by reducing pill burden and dosing complexity can help patients better manage their medications. Education and ongoing support are essential for enhancing patient understanding of their condition and the importance of adherence. Addressing financial barriers by implementing assistance programs can ease the financial strain, making medications more accessible. Lastly, enhancing communication between healthcare providers and patients fosters trust and allows for more personalized care, ultimately improving adherence and health outcomes for HF patients.^[97]^

##### 3.4.5.1. Dietary and lifestyle noncompliance in heart failure management

Adherence to dietary restrictions and lifestyle changes is critical in HF management. However, many patients struggle with these modifications, which are essential for improving both outcomes and QoL.

##### 3.4.5.2. Dietary noncompliance

Maintaining a low-sodium diet is one of the most significant challenges for HF patients. The high prevalence of sodium in processed foods, along with concerns over taste, makes adherence difficult. Educating patients on the harmful effects of sodium on heart health, providing practical tips for reducing intake, and offering ongoing support from healthcare providers are key strategies to improve compliance with dietary restrictions.^[101]^

##### 3.4.5.3. Lifestyle noncompliance

Engaging in regular exercise is vital for managing HF, but many patients face obstacles such as lack of motivation, physical limitations, or fear of worsening symptoms. Structured exercise programs tailored to individual needs, professional supervision, and peer-support can help overcome these barriers.^[102]^ Managing weight is crucial, as HF medications can contribute to fluid retention and metabolic changes. Offering dietary counseling, monitoring fluid intake, and promoting healthy eating habits can support weight management efforts.^[103]^

##### 3.4.5.4. Psychosocial factors in heart failure management

Psychosocial challenges, including depression, anxiety, and stress, significantly impact HF patients’ ability to engage in effective self-care practices. These emotional challenges can undermine motivation, leading to nonadherence to medication regimens and lifestyle changes.^[102]^

##### 3.4.5.5. Impact of emotional challenges

Patients experiencing depression may feel persistent sadness, fatigue, and a lack of interest, all of which can reduce motivation to follow treatment plans. This often leads to poor adherence, worsened symptoms, and increased healthcare utilization. Anxiety can cause excessive worry, physical symptoms like palpitations, and fear of exacerbating HF symptoms, making it difficult for patients to cope with daily stresses. This can result in avoidance of social interactions and reduced participation in self-care activities. Chronic stress activates the body’s “fight or flight” response, increasing heart rate and BP, which are harmful in HF. Stress can lead to poor sleep, unhealthy coping mechanisms (overeating or substance use), and difficulty adhering to treatment recommendations.^[102]^

### 3.5. Evidence-based interventions to address performance gaps

Self-management interventions in patients with HF have a variable degree of effectiveness in reducing hospitalizations, morbidity, and mortality. The studies^[103–106]^ have weaknesses in study design, implementation, and reporting. The effectiveness of each component of self-care and various combinations need to be studied on a larger scale. Reducing hospitalization, morbidity, and mortality. The studies^[104–106]^ have weaknesses in study design, implementation, and reporting. The effectiveness of each component of self-care and various combinations need to be studied on a larger scale.

There is no standardized protocol for self-management interventions in HF patients. Though self-management interventions must be customized to each patient based on stage of HF, health status, comorbidities, knowledge of patients and caregivers, financial status, accessibility to cardiac care centers, and psychosocial support, guidelines for health care workers to optimize self-care are necessary. Each component of self-care must be optimized.

#### 3.5.1. Educating patients

Knowledge and awareness are essential aspects of chronic disease self-management (CDSM) for individuals with HF. Successfully managing the condition involves patients having a thorough understanding of their illness, which includes understanding the nature of HF, the importance of taking medications as prescribed, and making necessary lifestyle adjustments, and knowing how to identify and address symptoms. Despite improvements in medical education and resources, many HF patients still face substantial gaps in knowledge and awareness. These gaps can hinder their ability to effectively manage their condition. Signs and symptoms of HF are often mistaken for normal aging and thus overlooked, leading to late detection of the exacerbation of HF.^[96]^

Nurse-led educational interventions have been shown to be effective in improving knowledge, adherence to drugs, and self-monitoring among patients with HF, thereby reducing hospitalizations and morbidity. Educational sessions by nurses and physicians during hospitalization, before discharge, and a few weeks after discharge, either virtually or face-to-face with patients and their caregivers, have improved self-care.^[100]^ Empowering patients and involving them in customizing goals of self-care in each follow-up session, along with counseling and motivation to optimize medication adherence, monitoring, and identifying symptoms of exacerbation, is cost-effective in improving self-care and health-related QoL.^[107]^ The effectiveness and feasibility of these interventions must be studied on a larger scale, especially in rural areas and low-income countries.

#### 3.5.2. Home monitoring

Regular monitoring of vitals and symptoms is necessary without a doubt, but there is a need for a guideline to decide the frequency, intensity, and duration of monitoring that must be personalized to each patient. E-health/telehealth has made follow-up and monitoring easier, especially during the COVID-19 pandemic. Patients in remote villages who are unwilling to travel miles for a follow-up appointment can be monitored virtually. Studies show that telephone-based follow-ups and telemonitoring services are effective in reducing hospitalization and mortality.^[108]^ Patients with prior hospitalization and NYHA class III HF monitored via implanted pulmonary artery sensor (wireless) had fewer hospitalizations than patients receiving standard care.^[109]^ However, its effectiveness compared to noninvasive home monitoring is not clear, as there is no standardized guideline for home monitoring. Though monitoring using implantable devices helps identify the risk of exacerbation early and prevent it, it is useful only if it is followed up with an action to address the concerning finding. The feasibility of such comprehensive management for many HF patients is questionable.

It is uncertain whether continuous monitoring of vitals, weight, and symptoms in routine HF management will significantly improve the outcome. Since each patient has a different degree of knowledge and skill set to operate technology/monitoring devices, it is difficult to discern the reliability of such monitoring.^[108]^ There is a need to develop a more cost-effective intervention to improve monitoring, especially in underserved communities.^[110]^

Contemporary meta-analyses show telemonitoring & remote patient monitoring reduces HF hospitalizations, with inconsistent effects on mortality and QoL; programs succeed when measurements (weight, BP, symptoms) trigger protocolized clinical responses rather than passive tracking.^[111]^

Recent RCTs testing app + connected devices + feedback report improvements in symptoms and self-care behaviors compared with usual care; effects on hard outcomes are emerging and context-dependent.^[112]^ Pilot trials of digitally enabled community health worker (CHW)/coach support indicate reduced early readmissions and high acceptability, supporting hybrid human-tech models. Practical challenges include overload, alert fatigue, privacy/security, device loss, and digital divide issues (connectivity, literacy, language) limit real-world impact, especially in underserved settings; aligning team workflows and reimbursement is essential for scale.^[113]^

#### 3.5.3. Weight and nutrition

Obesity is an independent risk factor for developing cardiovascular disease. However, in patients with HF, weight loss and low BMI are poor prognostic factors, whereas weight stability indicates a favorable prognosis. Patients with low BMI develop sarcopenia, friability, and cachexia. Nutritional screening to identify at-risk patients is necessary and can be done by anyone with minimal training.^[114]^ It is important to develop a standardized screening tool and criteria for malnutrition in HF. At-risk patients need to be evaluated by a multidisciplinary team to assess the nutritional status and develop a comprehensive diet and exercise regimen with the patient. Although data on the effect of morbid obesity (BMI > 40 kg/m^2^) on the prognosis of HF is limited, bariatric surgery is advised to these patients to significantly lower weight and consequently many health risks. Weight loss is recommended for young HF patients with a BMI > 35 kg/m^2^. Guidelines to optimize weight loss in overweight patients are needed.^[115]^

Regular monitoring of iron studies and iron supplementation in cases of deficiency is recommended. It is unclear whether increasing dietary iron will help with HF management. Though micronutrient deficiency is associated with poor outcomes, the effectiveness of routine supplementation is yet to be studied.^[115]^

There is no concrete evidence dictating fluid restriction in patients with HF, but guidelines suggest fluid intake to be 1.5 to 2 L/d. Fluid intake depends on HF symptoms and weather. Elderly patients have a depressed function of thirst centers, so adequate fluid intake is necessary to prevent dehydration. The effectiveness of fluid restriction in reducing hospitalizations and mortality is not known. Like fluid restriction, there is minimal evidence to support restricting salt in the diet of patients with HF. Salt intake < 5 g/d is recommended, which is the same as for the general population. In patients with NYHA class III and IV HF, salt intake > 3 g/d was associated with increased hospitalization, but in patients with NYHA class I and II HF, salt intake < 2 g/d showed poor prognosis. The effect of salt restriction on hospitalizations is inconclusive. MoveStrong, an exercise program for patients with chronic disease, showed promising benefits in improving the QoL. Participants were given materials, 1-on-1 remote training sessions by a physiologist, and nutrition education sessions by a dietitian.^[116]^ Patients followed the exercises and were satisfied with the program. The feasibility of this program on a larger scale needs to be studied.

#### 3.5.4. Adherence

Many systematic reviews have concluded that the most effective way to ensure adherence is through continuous, comprehensive interventions. Effective methods to increase drug adherence include a multidisciplinary approach, education, counseling, reminders, involving patients in the decision-making process, addressing adverse effects, avoiding polypharmacy, and simplifying the regimen.

A pharmacy-based interdisciplinary intervention, which involves patients visiting the pharmacy once a week, receiving their medications in weekly doses, having trained pharmacists check for side effects and drug interactions, and collaborating with the doctor in the event of side effects or changes in vital signs, has significantly improved adherence compared to regular care.^[117]^ But this study was conducted in a small number of patients; its feasibility and effectiveness on a larger scale are not known.

#### 3.5.5. Psychosocial support

Many patients with HF struggle with depression and anxiety, which may affect other components of self-care like adherence, monitoring, and nutrition. Therefore, regular screening for depression and anxiety must be incorporated into routine care. In a study to determine the effectiveness of psychospiritual intervention for patients with HF, it was noticed that though it significantly improved the mental health of patients, many patients reported that it did not change the overall QoL. It would have been impactful if available at diagnosis, participants said.^[118]^

Cognitive behavioral therapy decreases depressive symptoms. Patients must be encouraged to practice meditation and yoga and develop stress relaxation methods.^[115]^ Involving family members or caregivers in self-management interventions can significantly improve the QoL.^[119]^ However, this can negatively impact caregiver burden and their mental health.^[120]^ The components of caregiver involvement, the extent of involvement, and its effect on patient and caregiver outcomes need to be studied on a larger scale. Dyadic (patient and caregiver) interventions can improve the self-care of patients with HF, but a proper study with clear protocol on the implementation of interventions and their impact on patient and caregiver outcomes is lacking.^[120,121]^

#### 3.5.6. Sleep

Adequate and quality sleep is necessary for HF patients’ overall well-being. Inadequate sleep can also affect other aspects of self-care, such as adherence, exercise, and daily routine activities. Therefore, it is important to include a screening questionnaire for patients and their partners to assess adequate sleep during follow-up visits. The reason for inadequate sleep must be evaluated and treated. Depression, anxiety, obstructive sleep apnea, and fluid overload are some of the many causes of insufficient sleep and daytime sleepiness. Benzodiazepines may help, but they pose a risk for tolerance and addiction. They may impact elderly patients’ cognition and mobility. Effects and long-term consequences of sleep medications in patients with HF have to be studied.^[115]^

#### 3.5.7. Comparative effectiveness of self-management delivery strategies

Technology-based vs traditional education. Across recent syntheses, home telemonitoring and app-enabled education generally reduce HF-related or all-cause hospitalizations versus usual care, but effects on mortality and QoL are mixed and heterogeneous by program design. A 2023 meta-analysis of home telemonitoring reported significant reductions in HF hospitalizations but variable effects elsewhere, highlighting the importance of protocolized actions in response to alerts.^[111]^ A 2024 meta-analysis likewise found fewer hospitalizations without clear mortality benefit, suggesting optimization of component features is needed.^[122]^ Noninvasive telemonitoring RCTs pooled through 2024 (n ≈ 13,000) confirm hospitalization reductions with wide heterogeneity.^[123]^ Conversely, narrative appraisals emphasize that weights/symptom checks alone rarely move outcomes unless linked to timely clinical action and patient-facing education.^[124]^

Individual (1-to-1) versus group/peer-supported interventions. Empowerment-based, interactive counseling outperforms didactic education for self-care (symptom perception/response) and can be cost-effective versus lecture-style formats (RCT, n = 236).^[125]^ Early trials of digitally enabled CHW or coach support (phone reminders, brief counseling) show feasibility and clinically relevant 30-day readmission reductions vs usual care, though larger trials are needed.^[113]^ Small group or peer-support formats (including messaging platforms) tend to improve engagement and self-efficacy in long-term conditions; in HF, group-delivered caregiver–patient models are under active evaluation and may be attractive for patients citing isolation or low motivation.^[126]^

##### 3.5.7.1. Which strategy for which patient?

For older patients, those with multiple comorbidities, limited digital literacy, or living in rural areas, low-tech approaches tend to be most effective. Nurse-led or CHW–supported monitoring, combined with regular phone check-ins, generally outperforms app-only interventions in maintaining adherence and ensuring continuity of follow-up.^[113]^

Patients who have been recently hospitalized or who present with more severe disease (NYHA class III–IV) benefit most from structured telemonitoring systems. When these programs include clear escalation protocols, they provide the greatest reductions in hospitalizations.^[111]^ In contrast, individuals with high self-efficacy and strong caregiver support can achieve better outcomes with app-based self-care platforms connected to monitoring devices. These approaches improve symptom control and self-management, particularly when automated feedback is integrated with periodic clinician review.^[112]^

### 3.6. Challenges and barriers to effective implementation

#### 3.6.1. Barriers to implementing self-care

Using Sandelowski and Barroso’s method, a qualitative meta-summary identified 21 barriers contributing to nonadherence. The factors contributing to nonadherence to self-care included the presence of comorbidities, a lack of decision-making skills, not prioritizing self-care, problems with symptom recognition, or when the implementation of self-care caused adverse effects. Other barriers include habits that are difficult to break, lack of motivation, sophisticated and time-consuming self-care exercises, and poor communication with healthcare professionals. Financial difficulties, such as income loss and high medical expenses, often prevent patients from participating in self-care activities. On the other hand, having financial stability helps patients better manage their chronic HF. The analysis revealed several important insights. First, patients often had trouble understanding their symptoms, frequently mistaking them for issues related to other existing health problems, medication side effects, or emotional reactions, which caused delays in seeking help. Second, a lack of clear information, uncertainty, and misunderstandings hindered their ability to properly care for themselves. Third, negative emotions and mental health issues further impeded their self-care efforts. Lastly, support from family, friends, or community organizations was vital for effectively managing HF.^[127]^

A lack of knowledge is one of the main reasons HF patients fail to practice self-care. Patients have reported that they lack understanding of the importance of follow-up and medication adherence, and they have limited opportunities to ask healthcare providers questions. They find it difficult to make appointments, and both patients and health care providers agree that the time allotted for each appointment is insufficient to explain and answer patients’ concerns. Although providers sympathize with patients’ uncertainty about their illness, they struggle to explain the complex disease process to patients who have unrealistic expectations.^[128]^ An ideal situation is 1 in which the patient is comfortable expressing their concerns, and health care providers listen and answer every concern and query, helping them understand the disease process and the importance of self-management. Research has shown that meaningful conversations between patients and healthcare providers significantly improve the QoL in HF patients.^[129]^ J. P. Kosmala-Anderson et al concluded in their study that training physicians in effective self-management support delivery can enhance patient-centered care and boost their confidence in providing support.^[130]^ S. Lawn et al suggest a combination of e-learning and face-to-face sessions to improve the support skills of providers.^[131]^

Lack of continuity and consistency is another barrier. Each time the patient visits the hospital, a different physician or nurse attends, often changing the medications. Patients receive conflicting advice and end up more confused than before. Following up with the same provider each time helps build a rapport, and patients are more likely to open up about their concerns and preferences. Another barrier that requires attention is the lack of coordination among GPs, specialists, and cardiac care nurses’ social workers.

In a busy hospital setting, lack of streamlined admission and discharge is a barrier. ERs admit HF patients where beds are available. Patients report that they don’t have access to the same providers who can answer their questions. Lack of beds often leads to hurried patient discharges, resulting in an inefficient transition from inpatient care to self-care. Patients often struggle to determine who to consult when their symptoms worsen or when they have questions. There is a lack of expert support after office hours. Primary care providers believe they lack knowledge and skills and require the assistance of specialists to manage advanced HF patients. The number of patients overwhelms cardiologists, leaving them with insufficient time to diligently address each patient. Providers believe that a key individual or individual must be in charge of coordinating patient care and treatment, but no health professional feels fit to take on this responsibility.^[132]^

Many patient-provider conversations tend to take on a narrower approach wherein the provider lays out instructions for taking medications, diet, exercise, and monitoring, and the patient is expected to follow them. The purpose of support in these situations is to manage the disease well via education and persuasion. Providers talk about involving patients in decision-making or empowering them, but the patients often have limited scope for participation. Patients are expected to decide and manage the disease in accordance with professional recommendations. This often ends up with patient dissatisfaction and noncompliance with self-care. As opposed to this, a broader approach focuses on helping the patient live well with their condition. This can be done by shifting the focus from the provider perspective to the patient perspective. Providers need to consider each patient’s circumstances, lived experiences, concerns, and view of QoL along with managing the disease. Asking for the patient’s opinion on disease and its management and having an honest conversation when they vary from the provider is beneficial in achieving the final goal. Empowering patients to set individual goals, supporting them to build the knowledge, confidence, and skills to identify and manage their challenges is an important component of a broad approach to patient care. While all this is ideal, it is difficult at the same time. All providers may not have the skills to achieve this, and not all patients are honest about their opinion. Adopting a broader approach may sometimes be contradictory to conventional methods; providers need to have flexibility, interpersonal skills, and confidence to make tricky judgment calls. In some situations, it may be beyond the scope of the patient-provider relationship and hospital policies.^[133]^ It is a constant struggle to maintain the balance between managing the disease and the patient’s challenges.

#### 3.6.2. Patient perspectives on self-management

Qualitative studies highlight that many patients value simplified regimens, culturally tailored education, regular 2-way communication with trusted clinicians, and caregiver involvement; perceived burdens (dietary restrictions conflicting with cultural foods, fear of exercise, mistrust of tech) drive nonadherence.^[134]^ Patients’ preferred outcomes often extend beyond readmissions to functional status, energy, and independence; mixed-methods work shows patients prioritize symptom relief and daily functioning when weighing trade-offs.^[135]^ Newer qualitative research after 2023 underscores barriers such as self-capacity, motivation, and health/system navigation; peer/community support and clear action plans are recurrent facilitators.^[136]^ Designing CDSM with patient-reported outcomes, co-created goals, and options for peer/group support can improve engagement, particularly for individuals reporting isolation, low literacy, or cultural friction with diet/exercise advice.^[137]^

### 3.7. Future directions and recommendations

#### 3.7.1. Research priorities

Given the financial strain hospitalizations and acute management of worsening HF patients impose, it is crucial to address performance gaps in the implementation of CDSM. Further studies should concentrate on enhancing our understanding of patient education strategies, identifying barriers faced by both patients and healthcare providers, and exploring new and innovative methods to educate patients using advanced and AI-powered digital health tools.^[135]^ These tools should be more user-friendly and available in vernacular languages to overcome patient barriers. The studies should also concentrate on developing a personalized approach to customizing a CDSM protocol for patients, with the aim of determining whether such a personalized approach enhances patient outcomes.

With long-term monitoring of CDSM-managed patients and follow-ups involving parameters like hospitalizations, QoL, and mortality rate, the efficacy and sustainability of CDSM programs can be gauged.^[115]^ Large and long-term studies can also be utilized in creating standardized home monitoring guidelines to identify symptoms accurately and allow more precise and accurate changes in medication or dose by the patient to overcome the patient barrier to decision-making.

#### 3.7.2. Policy and practice recommendations

Healthcare providers should emphasize the integrated approach to patient care and must adopt ways to make the patients more involved in the decision-making process. Healthcare systems should be set up in a way that digital health tools and other sources are easily available and approachable for all the patients.^[138]^ Healthcare professionals should get more involved in bridging the communication gap and providing essential health literacy to the patients.

Considering the healthcare burden and overworked physicians, nationwide policies should be made to incentivize CDSM programs, and healthcare providers should be reimbursed for investing resources in the implementation of CDSM programs and patient education. More resources and funding should be allocated towards research and studies that further our understanding, ultimately leading to a decreased healthcare burden and costs from acute hospitalizations.

#### 3.7.3. Implementation science perspectives

Despite efficacy signals in trials, translation falters without attention to costs, scalability, sustainability, and equity. An AHA 2024 scientific statement reviewing HF implementation trials notes frequent improvements in therapy uptake with behavioral nudges, multidisciplinary care, and digital strategies, but finds limited use of implementation frameworks and few equity-oriented endpoints; scalability and durability are rarely assessed.^[139]^

Telemonitoring and team-based care require upfront investment (staffing, devices, analytics). Programs that reduce readmissions can shift costs to outpatient care; value depends on payer incentives and local capacity.^[124]^ Interventions validated in specialized centers often underperform in low-resource or rural settings because of workforce limits, device maintenance, and variable digital literacy.^[139]^ Engagement typically wanes after initial months; embedding tools into routine workflows, providing feedback loops, and aligning reimbursement for education/remote monitoring are crucial.^[133]^ Emerging roadmaps argue for integrating pragmatic trials into delivery systems, using iterative adaptation and equity metrics (uptake across literacy/language/socioeconomic strata).^[140]^

## 4. Conclusions

HF self-management works when patient, provider, and system factors are aligned around clear, actionable care. Education, exercise, and targeted diet changes are beneficial; telemonitoring and digital tools reduce hospitalizations only when data trigger protocolized clinical actions, not passive tracking. Medication adherence is the persistent bottleneck, best addressed by simplifying regimens, continuous counseling, and timely feedback. Integrating patient perspectives simplified plans, culturally tailored education, caregiver involvement, shared decisions, and clear action plans should be standard. Implementation for the real-world matters: build for scalability, workflow fit, reimbursement for education/remote patient monitoring, and equity (literacy, language, connectivity). Deploy by phenotype: low-tech nurse/CHW support for older or low-literacy groups; structured telemonitoring with escalation for recently hospitalized or NYHA III–IV; app-connected self-care with clinician review for high self-efficacy patients. Next steps are pragmatic, equity-aware trials measuring durability and costs. The payoff: better symptoms and QoL, fewer hospitalizations, and more reliable, equitable care.

## Author contributions

**Conceptualization:** Sanjana S. Nelogal, Sweta Sahu, Tirath Patel

**Writing – original draft:** Sanjana S. Nelogal, Sai Teja Yedam, Srija Reddy Koppula, Hafsa Imtiaz, Pranay Shettywarangale, Basira Shah, Vadali Avinash, Akshita Bhalla, Sweta Sahu, Tirath Patel.

**Writing – review & editing:** Sweta Sahu, Tirath Patel.
